# Oral Cellular Neurothekeoma

**DOI:** 10.1155/2013/935435

**Published:** 2013-04-04

**Authors:** Nader Emami, Faisal Zawawi, Rania Ywakim, Ayoub Nahal, Sam J. Daniel

**Affiliations:** ^1^Department of Otolaryngology-Head and Neck Surgery, McGill University, Montreal, QC, Canada H3A 1A1; ^2^Department of Otolaryngology-Head and Neck Surgery, King Abdulaziz University, Jeddah 21589, Saudi Arabia; ^3^Department of Pathology, McGill University, Montreal, QC, Canada H3A 2B4; ^4^Department of Otolaryngology-Head and Neck Surgery, McGill University, Montreal Children's Hospital, 2300 Tupper Avenue, Montreal, QC, Canada H3H 1P3

## Abstract

Cellular neurothekeoma is known as a cutaneous tumor with uncertain histogenesis. Very little involvement of mucosal membrane has been reported in the literature so far. This is a case report of an intraoral lesion in a 15-years-old girl. Histopathologic evaluation showed a tumor-consists of spindle to epitheloid cells forming micronodules in a concentric whorled shape pattern. Tumor cells were positive for CD63, vimentin, and NKI-C3. Total excision was performed and no recurrence happened after 16-month followup.

## 1. Introduction

Neurothekeoma (NTK) is a generally accepted term for a rare type of benign dermal tumours originating from nerve sheath, first proposed by Gallager and Helwig in 1980 [1]. This entity was previously described and known as nerve sheath myxoma.

Cellular NTK was later identified as a subtype based on histopathological and immunohistological features [2, 3]. It is generally a cutaneous lesion, and mucosal involvement has been rarely reported. These numbers are even less in case of cellular variant [4].

This is a report of a cellular neurothekeoma of the oral cavity in a 15-years-old girl. 

## 2. Case Report

This study is approved by the Research Ethics Board of the Montreal Children's Hospital and exempted from a full review.

**Figure 1:**
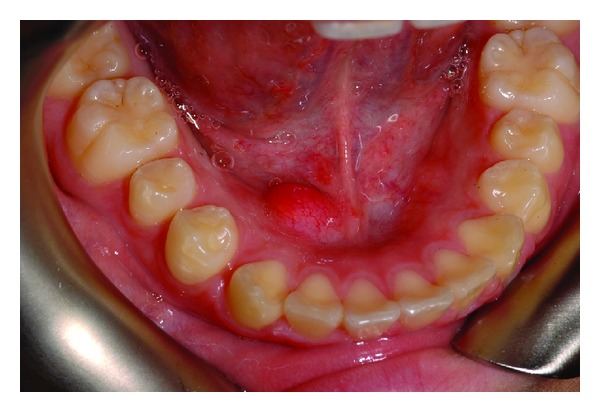
Intraoral submucosal lesion on the right side of the floor of the mouth of a 15-years-old girl, diagnosed with cellular neurothekeoma after biopsy.

A 15-year-old girl presented with a painless submucosal nodule on the anterior right side of the floor of the mouth measuring 0.8 cm in the largest dimension for an unknown duration (Figure 1). The patient had no other signs or symptoms.

Her past medical history included acute lymphoblastic leukemia (ALL), diagnosed at the age of 2 years requiring irradiation followed by bone marrow transplant. She has been in remission ever since. She also has a history of multiple acquired nevi which have been biopsied and returned benign.

An incisional biopsy was attempted to make the final diagnosis. The pathology report showed cellular NTK. A completion surgery was then performed.

There has been no sign of recurrence after 8 months of followup.

## 3. Histologic and Immunologic Evaluation

**Figure 2:**
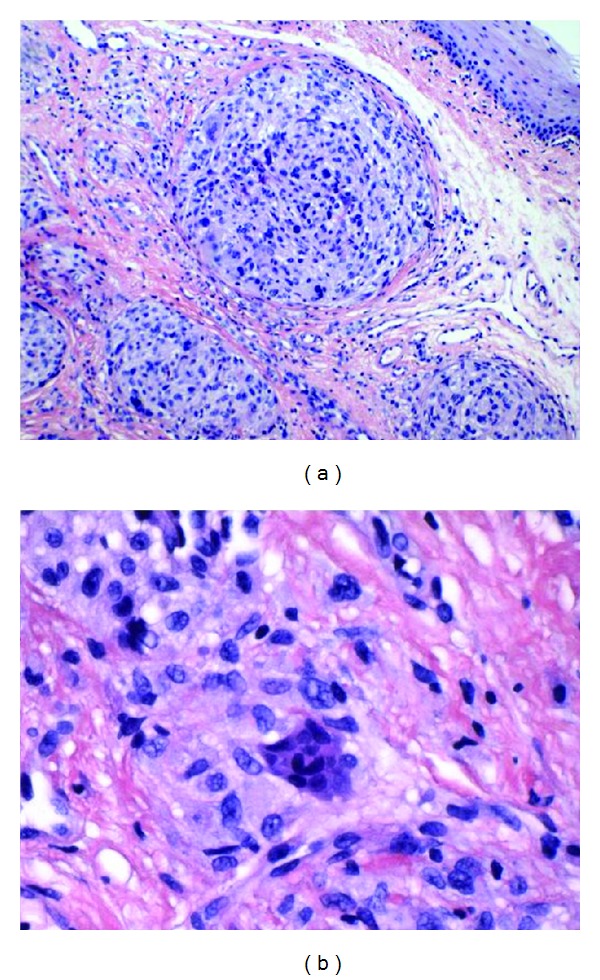
H&E stain, low (a) and high (b) power microscopic views. Intramucosal micronodules, shaped in a characteristic whorled pattern with concentric growth. Cells are epithelioid to spindle shaped with mild cytologic atypia.

The excised lesion measures 8 mm in the greatest dimension. Microscopic evaluation of hematoxyline-and-eosin- (H&E-) stained slides showed an unencapsulated neoplasm composed of spindle to epitheloid cells, arranged in well formed micronodules with concentric whorled shape appearance. Mucosa was widely involved and some invasion to submucosa was observed. Most of the neoplastic cells appeared fibroblastic and showed mild nuclear atypia. Osteoclast type giant cells were scattered within the whorled nodules. No myxoid matrix was seen and mitotic activity was almost absent (Figure 2).

Tumor cells strongly expressed CD63, vimentin, and NKI-C3, while desmin, smooth muscle actin (SMA), S100, CK7, CK20, CK5/6, epithelial membrane antigen (EMA), P63, CD1a, and MART-1 were all negative. The stromal dendritic cells were positive for Factor XIIIa and multinucleated giant cells were positive for CD10.

## 4. Discussion

Cellular NTK is usually a cutaneous lesion and rarely involves mucus membranes. Cases of oral mucosal involvement are even less likely to happen with only 5 reported cases in the English language literature [4].

Clinical differential diagnoses that we had in mind before biopsy for such lesion in a patient with a history of ALL were more common possibilities including lymphoma, minor salivary gland tumors (pleomorphic adenoma), and granular cell tumor. 

Melanocytic tumor is usually included in the differential diagnosis of NTK especially in high risk patients like the present case with the history of irradiation. Although there are histological similarity and immunological overlap between these neoplasms, such as spindle and epitheloid cells in whorling pattern and the presence of NKI/C3, NTK is generally negative for protein S100 and can be differentiated from melanocytic lesions.

Our case had all the histopathologic features of cellular NTK including the presence of epitheloid and spindle cells in the setting of whorled nodules, absence of myxoid matrix, and occasional presence of osteoclast-like giant cells [5]. These findings together with the findings from immunostain studies confirmed the diagnosis of cellular NTK.

Presence of multiple nevi has been reported in previous series as well as our case, but there is no enough evidence to count it as a risk factor because of small the number of cases.

Recurrence rate has been reported to be between 3 and 15% in NTK and seems to be related to mitotic activity within tumor cells, and most of the recurrences were observed in cases with incomplete excision [5, 6].

This case was reported to raise awareness about an unusual presentation of neurothekeoma as it seems to be underreported in the literature [5].

## Abbreviations

NTK: Neurothekeoma
ALL: Acute lymphoblastic leukemia
H&E: Hematoxyline and eosin
EMA: Epithelial membrane antigen
SMA: Smooth muscle actin.
